# Outcome Measures in Clinical Trials of Patients With Myasthenia Gravis

**DOI:** 10.3389/fneur.2020.596382

**Published:** 2020-12-23

**Authors:** Jan Lykke Scheel Thomsen, Henning Andersen

**Affiliations:** Department of Neurology, Aarhus University Hospital, Aarhus, Denmark

**Keywords:** myasthenia gravis, classification, clinical trials, review, outcome measure, rating scale

## Abstract

Myasthenia gravis (MG) is a heterogeneous disorder whose clinical presentation ranges from mild ocular deficits to severe widespread weakness. This variance poses a challenge when quantifying clinical deficits. Deficits and symptoms are quantified using standardized clinical scales and questionnaires which are often used as outcome measures. The past decades have seen the development of several validated outcome measures in MG, which are used in clinical trials to obtain regulatory approval. In recent years, emphasis has moved from objective assessments to patient-reported outcomes. Despite a growing body of literature on the validity of the MG-specific outcome measures, several unresolved factors remain. As several novel therapeutics are currently in clinical development, knowledge about capabilities and limitations of outcome measures is needed. In the present paper, we describe the most widely used clinical classifications and scales in MG. We highlight the choice of outcome measures in published and ongoing trials, and we denote whether trial efficacy was reached on these outcomes. We discuss advantages and limitations of the individual scales, and discuss some of the unresolved factors relating to outcome assessments in MG.

## Introduction

Myasthenia gravis (MG) is an autoimmune neuromuscular disease characterized by fatigable muscle weakness due to autoantibodies targeting components of the neuromuscular junction (1). Symptoms and deficits involve ocular, bulbar, respiratory and proximal limb muscles, and they fluctuate in a diurnal and day-to-day pattern. This fluctuating nature of symptoms challenges assessments of disease severity. Deficits and symptoms are measured using validated clinical scales. The past decades have seen the development of several clinical scales reflecting objective, patient-reported and composite measures of disease severity. These validated outcome measures are frequently employed as primary and secondary efficacy parameters in randomized controlled trials (RCT). Several RCTs of currently used immunosuppressants have produced ambiguous results concerning their efficacy in MG. This lack of efficacy may be due to trial-related factors, including sample size issues (e.g., low recruitment), design (e.g., length and inclusion criteria) and insufficiently sensitive outcome measures (e.g., floor and ceiling effects) (2). Accordingly, the current use of these treatments is based on expert consensus and convincing efficacy in daily clinical use. Treatment of MG has recently entered a new era with the development of monoclonal antibodies targeting specific pathophysiologic culprits. As RCTs of these therapeutics may lead to regulatory approval of new treatments, knowledge of the capabilities and limitations of the clinical scales is imperative in understanding the efficacy of current and future treatments in MG.

## Clinical Classification

MG is a heterogeneous disease with several possible classifications according to disease and patient-related factors (1). Type of autoantibody enables classification accordingly, which may directly affect treatment choice. Age at onset enables classification into early-onset and late-onset disease; the former having a female predominance and a higher frequency of thymic hyperplasia. Symptom distribution may be used to classify MG into ocular and generalized MG; and MG may be classified by presence or absence of thymoma.

Although subpopulations of MG are distinguishable, patients are often classified according to the severity of deficits using the Myasthenia Gravis Foundation of America (MGFA) Classification. In 2000, the MGFA Classification was defined as an iteration of previously used classifications (3–6). Patients are classified according to level of overall severity, spanning ocular-only (I), mild (II), moderate (III), severe (IV) and intubation (V), with additional subclassification related to axial/extremity (a) or bulbar (b) predominance. The MGFA Classification is not a recommended outcome measure owing to its poor correlation with summated rating scales (7, 8) and high dependence on physician interpretation. The MGFA Classification is a system broadly characterizing patients according to severity of disease and prognosis.

## Outcome Measures

In the 1930s, the use of ephedrine (9), acetylcholine esterase inhibitors (10), pituitary extract (11) and thymectomy (12) enabled non-quantifiable individual-level descriptions of treatment-related improvements in MG. A rating scale specific to MG was not introduced until the 1980s, and the subsequent decades saw the development of several MG-specific clinical scales (Table 1). Several publications review the various measures in detail (13, 25). Currently, the QMG, the MGC, the MG-ADL, and the QOL15(r) are the most widely used scales in clinical trials. Recently, the MGII was developed and has several potential advantages, however this scale has not been used in any clinical trials yet. Accordingly, the QMG, the MGC, the MG-ADL, and the QOL15(r) will be described below. The MGII will be discussed in context of advantages and limitations of these scales.

**Table 1:** MG-specific outcome measures.

**Name**	**Year**	**Type**
Myasthenia muscle scale (13)	1983	Objective
MG score (14)	1987	Objective
Basta neurologic institute rating scale (5)	1988	Composite
Quantitative myasthenia gravis (15)	1998	Objective
MG activity of daily living (16)	1999	Patient-reported
MG questionnaire (17)	2002	Patient-reported
MG manual muscle test (6)	2003	Objective
Ocular-bulbar-facial-respiratory scale (18)	2006	Objective
MG composite (19)	2008	Composite
MG quality of life (20)	2008	Patient-reported
MG quality of Life 15 items (revised) (21, 22)	2008 (2016)	Patient-reported
MG disability scale (23)	2014	Patient-reported
MG impairment index (24)	2016	Composite

The Quantitative Myasthenia Gravis (QMG) scale was introduced in 1998, serving as an objective measure of disease severity (16). The QMG encompassed eight items in the first version (26). It was later expanded to include 13 items (15). In a subsequent revision, the patient-reported items were replaced by physician examinations resulting in its current version (16). The QMG assesses muscle strength and fatigability using objective measures of double vision, ptosis, facial muscles, dysphagia, dysarthria, proximal limb, hand muscles, neck muscles and respiratory function. These assessments are somewhat time consuming and require equipment. Accordingly, in daily clinical practice use of the QMG is challenging. Each item is given a score of 0–3, resulting in an unweighted total score of 0–39. A higher score corresponds to more severe disease. Based on data from the cyclosporine trials (15, 16, 27), a 3-point change is considered clinically meaningful, with a modification in milder cases where a 2-point change is considered sufficient (13). Reliability is high and interobserver variability is low (16, 28, 29).

The MG Activity of Daily Living (MG-ADL) scale is a patient-reported outcome developed in 1999 (17) as a quickly administered set of questions examining frequency and severity of key MG symptoms. The MG-ADL was constructed as an expanded version of the patient-reported sub-items from another scale (15). Using a recall period of a few weeks, eight questions assess ocular function, speech, chewing, swallowing, respiratory function, and strength of proximal upper and lower extremities. Each item is scored from 0 to 3, which results in an unweighted total score of 0–24 points. A higher score indicates more severe symptoms. Based on a longitudinal study on the MG-ADL, the QOL15 and the physician impression of change (30), a 2-point change is considered clinically meaningful. Reliability is high (30).

The MG Composite (MGC) scale was developed in 2008 (20). It was constructed using the top performing items of the QMG, the MG-ADL and the Manual Muscle Test during a trial of mycophenolate. Six physician-assessed examinations evaluate ocular, neck and proximal limb muscles. Furthermore, four patient-reported items assess speech, chewing, swallowing and respiratory function. All patient-reported items are from the MG-ADL. A group of MG experts decided on item-score weighting based on symptom severity. Total score spans from 0 to 50; a higher score indicating more severe disease. A 3-point change is considered clinically meaningful based on physician's impression of change (31). The MGC has been reported to have a high reliability (31).

The MG Quality of Life 15-items (QOL15) was developed in 2008 as a patient-reported outcome (22). It was based on a large 60-item MG questionnaire (21). The current 15 questions were based on feedback from patients and on responsiveness of the individual items during a trial of mycophenolate. Using a recall period of a few weeks [originally 4 weeks (22)]), these 15 questions assess ocular symptoms, swallowing, speech, proximal limb function, mobility, personal grooming, work, social life, activities, fluctuations and psychological items. Scoring is qualitative. Each question is scored from 0 to 4, resulting in a total score in the range of 0–60; a higher score indicates poorer quality of life. The QOL15 score was slightly revised to its present version during subsequent international validation (23). The QOL15r retains the original 15 questions using a slight re-phrasing of some items and reducing the item score to a range of 0–2. Reliability is high (23, 32). The responsiveness has not been studied or published. The questionnaire has been validated in various languages and cultures.

## Outcome Measures in Published and Ongoing MG Trials

Choice of primary and secondary endpoint(s) vary among the published and ongoing RCTs. In Table 2, trials with more than 30 participants are summarized, and their results are denoted according to the prespecified analysis.

**Table 2:** RCTs in MG with ≥30 participants and available results.

**Year**	**Trial**	**Primary Endpoint**	**Secondary Endpoint**
1993	Cyclosporine (26)	MG Score* Steroid-sparring Antibody-titer*	Treatment failures
1997	IVIG vs. plasma exchange (32)	MMS	Antibody titer Time-to-effect
1998	Azathioprine (add-on to prednisone) (33)	Steroid-sparing* Treatment failure* Duration of remission*	Muscle strength (handheld dynamometry, walking time, swallowing time, forced vital capacity, subjective scoring)
2005	IVIG 2 vs. 1 g/kg for exacerbation (34)	MMS	Time-to-treatment response Forced vital capacity Antibody titer Intubation or nasogastric tube
2007	IVIG (35)	QMG*	SF-EMG RNS Post-intervention status*
2008	Mycophenolate (add-on to prednisone) (36)	QMG	MMT MG-ADL Forced vital capacity* SF-36 Treatment failure Global assessment of response Antibody type
2008	Mycophenolate (37)	Treatment response (post-intervention status steroid sparing effect, pyridostigmine dose)	Steroid sparing Pyridostigmine dose QMG SF-36 MG-ADL Global Assessment of Severity Antibody titer
2011	IVIG vs. plasma exchange (38)	QMG	SFEMG Post-intervention status Antibody titer
2011	Tacrolimus as steroid-sparing agent (39)	Steroid-sparing	QMG MG-ADL
2016	Methotrexate as steroid-sparing agent (40)	Steroid-sparing	QMG MG-ADL MMT QOL15 MGC*
2016	Thymectomy in non-thymomatous MG (41)	QMG* Steroid-sparing*	Treatment-associated symptoms* SF-36 MG-ADL* Post-intervention status* Use of immunosuppressants*
2017	Eculizumab in refractory MG (Phase 3) (42)	MG-ADL	QMG* MGC QOL15*
2017	Tacrolimus (43)	QMG	MGFA Classification MG-ADL MMT Steroid sparring
2018	Rituximab^1^ NCT02110706	Steroid-sparing effect	MGC QMG
2019	Efgartigimod (Phase 2) (44)	Safety	MG-ADL* QMG* MGC QOL15r*
2019	Rozanolixizumab (Phase 2) (45)	QMG	MGC MG-ADL*
2019	Iscalimab (Phase 2)^1^ NCT02565576	QMG	MGC MG-ADL QOL15
2020	Zilucoplan (Phase 2) (46)	QMG*	MG-ADL* MGC* QOL15r
2020	IVIG (Phase 2)^1^ NCT02473965	Steroid-sparing effect	
Ongoing	Salbutamol (Phase 2/3)^1^ NCT03914638	QOL15	MG-ADL QMG MGC NeuroQOL
Ongoing	Ravulizumab (Phase 3)^1^ NCT03920293	MG-ADL	QMG QOL15r
Ongoing	Efgartigimod (Phase 3)^1^ NCT03669588	MG-ADL	QMG
Ongoing	Rozanolixizumab (Phase 3)^1^ NCT03971422	MG-ADL	MGC QMG MG Symptoms PRO
Ongoing	Zilucoplan (Phase 3)^1^ NCT04115293	MG-ADL	QMG
Ongoing	Rituximab^1^ NCT02950155	QMG & steroid-sparring effect	QMG MG-ADL MG-QoL

Ongoing phase 3 RCTs in MG^1^. Statistical significance according to prespecified analysis is denoted by ^*^.

1 *Accessed 17th of August 2020 on ClinicalTrials.gov*.

*MMS, Myasthenia muscle scale; QMG, Quantitative Myasthenia Gravis; MMT, Myasthenia Gravis Manual Muscle Test; MG-ADL, Myasthenia Gravis Activities of Daily Living; QOL15, Myasthenia Gravis Quality of Life 15-items; SF-EMG, Single-fiber electromyography; RNS, Repetitive Nerve Stimulation; MGFA, Myasthenia Gravis Foundation of America*.

The prespecified endpoints have not been reached in several trials (Table 2). This may be due to a lack of efficacy; however, lack of efficacy may also result from sample-size issues, trial design and choice of statistical analysis.

Prior to 2017, the primary endpoint was mainly objective assessments (15, 27, 33, 35–37, 39, 42), antibody titers (15, 27) and the steroid-sparing effect (27, 34, 38, 40–42). The REGAIN trial evaluating eculizumab (43) was published in 2017 and was the first trial to introduce the MG-ADL as a primary endpoint. Currently, most ongoing phase 3 trials rely on the MG-ADL as a primary endpoint (Table 2). A trial of rituximab applies a composite measure of QMG and steroid-sparing effect, and a trial of oral Salbutamol is using the QOL15 as primary endpoint. Recently, the QMG is mostly used as a secondary endpoint in phase 3 trials (Table 2) and as a primary endpoint in pilot studies and phase 2 trials, including trials of mycophenolate (2003) (47), terbutaline (2009) (48), eculizumab (2013) (49), belimumab (2018) (50), rozanolixizumab (2019) (46), iscalimab (2019), and zilucoplan (2020) (51).

## Advantages, Limitations and Unresolved Factors

In recent years, the regulatory authorities have emphasized the use of patient-reported outcomes as primary efficacy parameter in clinical trials. Accordingly, several ongoing trials in MG use patient-reported outcomes as primary endpoint (Table 2). Symptoms fluctuate in MG; hence, objective assessments may not necessarily reflect patients' experienced symptom burden. Consequently, patient-reported outcomes are preferred as primary outcomes in MG trials.

Few patient-reported scales have been developed in MG (Table 1). The MG-ADL is validated, it has been tested in several trials, it is quick and easy to administer, and it assesses disease severity using questions specifically addressing MG symptoms. However, several symptoms of MG are not assessed, and the negative consequences of treatment (e.g., side-effects) are not addressed. Despite improvements in symptoms during treatment, the overall quality of life may be more severely affected due to, e.g., intolerable side-effects. Therefore, health-related quality of life measures may be considered more relevant outcome parameters. Using the QOL15 score introduces new challenges as factors unrelated to MG symptoms may affect quality-of-life scores (22, 52–55). Hence, relying on the QOL15 as primary endpoint may result in inadequate power to detect improvements in core MG-related symptoms. This may, in turn, result in issues relating to adequate trial recruitment. Improvements in the QOL15(r) score should therefore be considered as supplementary information when using the MG-ADL as primary endpoint. The use of a single patient-reported question assessing perceived degree of normal (Single Simple Question, SSQ) (56) has shown a high degree of correlation with the QOL15 and other MG measures, however this has not been tested prospectively. The Myasthenia Gravis Impairment Index (MGII) (57) is a newly developed composite outcome measure consisting of patient-reported items and physical examinations. The patient-reported subitems have excellent reliability as a stand-alone scale (57), however responsiveness and clinical meaningful change has only been published on the composite measure (58).

Some MG symptoms are poorly reflected by the MG-ADL. Neck weakness is not addressed although it is a debilitating symptom in some patients. Assessment of limb muscle fatigability is restricted to few shoulder and hip activities, although fatigability is one of the most relevant symptoms in patients with MG (59) potentially affecting several ADL functions. The QMG scale specifically addresses both complaints. The QMG is a well-established test providing evidence of responsiveness during various treatments; however, the QMG may be more sensitive to changes in ocular, limb and axial muscles than to changes in bulbar and respiratory functions (60). Thus, the QMG provides valuable objective information complementing the patient-reported outcomes, however objective assessments of respiratory and bulbar functions are still lacking.

MG symptoms contribute differently to the degree of clinical disability. Obviously, respiratory failure is more medically severe than persistent ocular symptoms. Hence, weighted scores as used in the MG-Composite may capture more clinically relevant information concerning disease severity. Thus, the MG-Composite may serve as an alternative to linear disease measures, complementing both the patient-reported outcomes and the QMG.

Degree of clinical disability is heterogeneous; hence, clinical scores should cover the entire spectrum ranging from mild to severely affected cases. However, there is considerable floor-effect in the MG-ADL (61) limiting its use in milder cases. The MGII shows less floor-effect than both the MG-ADL and the MGC (57), and it was recently shown to provide clinically relevant supplementary information to the MG-ADL (61). Interestingly, the MGII correlates only moderately with the QMG and the QOL15 during follow-up (58). Until now, the MGII has not been used as an outcome measure in trials, but it has the potential as an attractive alternative to other secondary outcomes. Due to the emphasis by regulatory authorities on patient-reported outcomes, the MGII is currently best suited as a secondary endpoint. MGII may enable superior assessment of efficacy covering a larger spectrum of disease severity if used as a primary endpoint, however this remains to be studied in RCTs. Further, the responsiveness and clinical meaningful change of the MGII patient-reported items as a stand-alone scale is unsettled.

Response to treatment is variable, and the overall treatment-effect consists of patients with both minor and larger improvements. Accordingly, the point-change required for a clinically meaningful improvement has been established on the MG-ADL, the QMG, and the MGC. This enables responder-analysis and assessments of clinical meaningful effects while negating minor placebo-effects and natural fluctuations. The pooled QMG response of several RCTs (62) detected significant effects over placebo on both continuous and categorical analysis. The MGFA Post Intervention System (MGFA-PIS) apply this required point-change on the MGC (recommended) or the QMG in order to address whether patients improve or deteriorate (2). Only few studies have applied the MGFA-PIS as an outcome measure (Table 2), however assessments or minimal manifestation and clinical remission are also included in the MGFA-PIS. Recently, to obtain patient acceptable symptom states (Patient Acceptable Symptoms Score, PASS) the cut-off values required on several clinical scales (the QMG, the MGC, the MG-ADL, the QOL15 and the MGII) were analyzed (63). It is currently unsettled whether dichotomized assessments of minimal manifestation or PASS is feasible in clinical trials.

No prospective study has analyzed the relations between the four most frequently used scales (the QMG, the MGC, the MG-ADL and the QOL15). Correlations between some of the scales have previously been published (30–32, 57, 58, 64), and the relations between objective (QMG and MGC) and patient-reported measures (MG-ADL and QOL15) seem attenuated during treatment and follow-up. One study (58) has applied the QMG, the MGC, the MG-ADL and the QOL15 to the same population; however, between-scale correlations were not published. It is unknown whether improvements on objective scores are accompanied by equal improvements on patient-reported outcomes (e.g., MG-ADL and QOL15/QOL15r).

There is a lack of information concerning how outcome measures are affected by basic patient characteristics and how the scales perform in various subpopulations. Such information is crucial in design of clinical studies, and it is critical when determining relevant change in burden of symptoms and deficits during routine care. Sex differences characterize early and late-onset subpopulations of MG; hence, females often have longer disease duration than males. Further, studies report sex differences in rates of refractory MG (65–67). Most recent and ongoing trials focus on severe or refractory patients; hence, trial populations may consist mainly of females, and participants may have long-standing disease. It is unsettled whether sex and disease duration affect potential for improvement on current outcome measures, and it is unknown whether current outcome measures are equally applicable in the various MG subpopulations.

When applying the current outcome measures, a major challenge is inability to capture all clinically relevant factors in MG. Fatigue is a relevant feature of MG in addition to muscular fatigability (68). Being a subjective feeling of exhaustion, fatigue is preferably quantified using patient-reported outcomes. Several generic fatigue scales have been used in MG, including the Neuro-QOL Fatigue Scale (68) and the Chalder Fatigue Scale (53). Only the REGAIN trial included fatigue as a secondary outcome (69). Although the QOL15 was not designed to specifically incorporate fatigue, a high degree of correlation has been established between fatigue and QOL15 (53, 69). This suggests some responsiveness to improvements in fatigue in addition to MG specific symptoms. Further, the patient-reported subitems of the MGII incorporate fatigue (57). Change-correlations between the MGII and the Neuro-QOL Fatigue Scale are moderate and equally directed (58). Whether fatigue scores complement improvement captured by the QOL15 or MGII scores remains to be studied.

Use of treatment as well as presence and severity of side effects are not systematically assessed in any of the outcome measures despite their clinical relevance. Steroids are frequently used during MG exacerbations and as effective bridging therapies when tapering immunosuppressive agents. Some patients require chronic steroid therapy due to inadequate symptomatic control. Several trials have used the steroid-sparing effect as an outcome measure (Table 2). Due to the side-effect profile of chronic steroid exposure, a reduced steroid dose is equated to improvement on MG scales. Reduction in other therapies (e.g., pyridostigmine or immunosuppressive agents) or a change in therapy (e.g., intravenous to subcutaneous immunoglobulin) may result in better quality of life despite stability in MG symptoms; however, this is only indirectly assessed by sub-items of the QOL15(r) and not addressed by any of the symptom-orientated scales. Risk of side effects may result in significant psychological stress, especially when considering cancer risk in young patients requiring long-term treatment or potentially teratogenic effects in fertile woman. Since MG is a chronic disease usually requiring treatment for decades, treatment satisfaction may be considered as important as symptomatic control. Treatment satisfaction is not systematically assessed using any of the current outcome measures.

In coming years, the use of tele-medicine will likely increase, especially due to the current global pandemic when monitoring immunocompromised patients. Further, virtual care may increase patient willingness to participate in RCTs owing to fewer physical attendances. Accordingly, validated measures assessing MG functioning through virtual care are needed. It is unsettled how the current MG scales function in a virtual setting. Some objective assessments are feasible, especially of ocular and bulbar involvement, however pure patient-reported measures will likely result in the most robust assessments. This area currently merits further research.

Patient-reported outcomes are often used as primary endpoints in establishing efficacy of novel treatments. Several of the recent trials focus on medically severe and refractory patients. However, a large proportion of patients are mild to moderately affected. New therapeutic options are warranted addressing unmet medical needs in this large group of patients. None of the current patient-reported outcomes enables detection of improvement on the entire severity continuum. In addition, no single patient-reported scale captures both the quantitative and qualitative aspect of improvement in MG symptoms during treatment.

## Conclusions

Several MG-specific outcome measures have been developed, reflecting objective disease burden, patient-reported symptom severity and health-related quality of life. Each scale has distinct advantages relating to MG assessments and complements information obtained from other outcome measures. Detailed assessments of treatment efficacy should currently incorporate patient-reported assessments (e.g., MG-ADL), quality-of-life measurements [e.g., QOL15(r)], objective assessments (e.g., QMG) and composite measures (e.g., MGC or MGII). Fatigue measures (e.g., NeuroQOL) may provide additional and relevant information. However, several clinically relevant issues are not addressed by any of the current scales, and the relation of several basic patient characteristics to current outcome measures remain unsettled. This restricts thorough assessment of treatment efficacy and may limit conclusions concerning validity across subpopulations in MG.

## Author Contributions

JT wrote and revised the manuscript. HA co-authored and revised the manuscript for intellectual content. Both authors contributed to the article and approved the submitted version.

## Conflict of Interest

JT has received speaker honorarium from Alexion. HA has received research support from Sanofi Genzyme and CSL Behring, and received travel support and speaker honoraria from Novo, Alexion, Sanofi Genzyme, Octapharma, and CSL Behring and served as consultant on advisory board for NMD Pharma.
